# Miscellaneous

**Published:** 1887-06

**Authors:** 


### MISCELLANEOUS.

WRINKLES.—Wrinkles are due to the gradual wearing away of flesh underneath the cuticle. Why does it wear away ? Because the facial muscles have either too little or the wrong kind of exercise. It will be observed that wrinkles usually take a downward course. This is due to the wrong kind of exercise. What exercise ? Why, the washing and wiping of the face, to be sure. Reverse the process, and, instead of rubbing the face down in washing and wiping, always rub upward. This will have the effect of counteracting the tendency of the flesh to depart from under the cuticle, and will keep the face free from wrinkles. It is rather an awkward habit to acquire at first, but perseverance will make it second nature, and the result is worth many pains. This exercise is designed particularly for the benefit of the eyes and the upper portion of the cheeks. Then, for the middle and lower portion of the face, where hollowness rather than wrinkles is often noted, another plan must be taken. The facial muscles are subjected to very slight activity in the ordinary exertions of eating and talking. To fill the cheeks out round and plump it is necessary to develope the muscles there. These muscles are very slight at the best, and any special effort well directed will increase them in capacity and size. An excellent exercise for this purpose is this : Take a piece of soft leather—kid or chamois skin will do —and put the end of it between the teeth ; then chew gently upon it for several minutes, taking care not to raise the teeth from the leather. If the teeth are raised it will bring into play only the ordinary muscles of mastication, whereas the purpose is to develope those that are seldom used. One who tries this method will find the cheek going through a queer action that is anything but graceful and pretty ; nevertheless, it is immensely effective and will restore to its youthful plumpness even the most hollow cheek. Try it faithfully and you will be convinced.

A Mania for Dosing.—It is about time to organize a movement for preventing the intemperate use of "temperance drinks." The manner in which mineral waters, acid phosphate, "nerve food," and "lactart" are swallowed at all hours of the day, and by all sorts of men, with no real knowledge of their nature or effects, is quite astonishing, and in many casas, no doubt, almost as pernicious, as the habit which it in a measure supercedes—the taking of the matinal cocktail, the midday beer, and the postprandial wine or spirits. Much of this guzzling is due to the mania for dosing, which is almost a national characteristic. If, owing to improper eating or any other cause, a man "doesn't feel just right," the first thing he does is to take a drink of something, hit or miss, while, if he "feels bad," a dose of patent medicine, or some other nostrum, follows. Some of the preparations are, perhaps, harmless, but others are obviously of so potent a nature that they should be used sparingly, and commonly only upon the advice of a physician. To burn the stomach with acids, or purge the bowels with mineral drinks, in the hap-hazard manner often indulged in, is to trifle recklessly with the health. For a person in an approximately normal condition there is no need of either stimulants, tonics, "nerve foods," purgatives, or other disturbers of nature. Good, plain food, fruit in abundance, milk, eggs, with a moderate supply of water that is cool, without being iced, or tea and coffee for those who must have "something else," constitute a summer regimen that is not improved by dosing of any sort.

---

We are always glad to hear good accounts of the remedies advertised in our columns. Indeed, it is our invariable custom to reject all offers of advertising of nostroms or wares, which do not commend themselves to our judgment as conducive to the health, comfort or enjoyment of those who employ them. The following testimonial needs no further introduction:

Mt. Hamill, Ia., April 4, 1887.

The Provident Chemical Works, St. Louis, Mo.

Gentlemen:—Yours and sample of Crystalline Phosphate to hand. Am pleased with its appearance, its compact form must commend itself to every physician. Shall be pleased to use it where indicated, and continue it if equal to other phosphates, as it certainly is handier and cleanlier. Yours Truly,

C. F. Wahrer, M. D.

